# 3D‐Printed Osteoinductive Polymeric Scaffolds with Optimized Architecture to Repair a Sheep Metatarsal Critical‐Size Bone Defect

**DOI:** 10.1002/adhm.202301692

**Published:** 2023-09-17

**Authors:** Charlotte Garot, Sarah Schoffit, Cécile Monfoulet, Paul Machillot, Claire Deroy, Samantha Roques, Julie Vial, Julien Vollaire, Martine Renard, Hasan Ghanem, Hanane El‐Hafci, Adeline Decambron, Véronique Josserand, Laurence Bordenave, Georges Bettega, Marlène Durand, Mathieu Manassero, Véronique Viateau, Delphine Logeart‐Avramoglou, Catherine Picart

**Affiliations:** ^1^ CNRS EMR 5000 Biomimetism and Regenerative Medicine (BRM) INSERM U1292 Biosanté CEA Université Grenoble Alpes 17 avenue des Martyrs Grenoble F‐38054 France; ^2^ Ecole Nationale Vétérinaire d'Alfort Université Paris‐Est Maisons‐Alfort F‐94704 France; ^3^ CNRS INSERM ENVA B3OA Université Paris Cité Paris F‐75010 France; ^4^ INSERM Institut Bergonié University of Bordeaux CIC 1401 Bordeaux F‐33000 France; ^5^ CIC‐IT INSERM Institut Bergonié CHU de Bordeaux CIC 1401 Bordeaux F‐33000 France; ^6^ INSERM U1209 Institute of Advanced Biosciences Grenoble F‐38000 France; ^7^ Institute of Advanced Biosciences Université Grenoble Alpes Grenoble F‐38000 France; ^8^ Service de Chirurgie Maxillo‐Faciale Centre Hospitalier Annecy Genevois 1 avenue de l'hôpital Epagny Metz‐Tessy F‐74370 France; ^9^ Institut Universitaire de France (IUF) 1 rue Descartes Paris CEDEX 05 75231 France

**Keywords:** 3D printing, bone tissue engineering, large animal models, medical devices, surface coatings

## Abstract

The reconstruction of critical‐size bone defects in long bones remains a challenge for clinicians. A new osteoinductive medical device is developed here for long bone repair by combining a 3D‐printed architectured cylindrical scaffold made of clinical‐grade polylactic acid (PLA) with a polyelectrolyte film coating delivering the osteogenic bone morphogenetic protein 2 (BMP‐2). This film‐coated scaffold is used to repair a sheep metatarsal 25‐mm long critical‐size bone defect. In vitro and in vivo biocompatibility of the film‐coated PLA material is proved according to ISO standards. Scaffold geometry is found to influence BMP‐2 incorporation. Bone regeneration is followed using X‐ray scans, µCT scans, and histology. It is shown that scaffold internal geometry, notably pore shape, influenced bone regeneration, which is homogenous longitudinally. Scaffolds with cubic pores of ≈870 µm and a low BMP‐2 dose of ≈120 µg cm^−3^ induce the best bone regeneration without any adverse effects. The visual score given by clinicians during animal follow‐up is found to be an easy way to predict bone regeneration. This work opens perspectives for a clinical application in personalized bone regeneration.

## Graphical abstract

A new osteoinductive medical device combining a 3D‐printed cylindrical scaffold made of polylactic acid with a surface coating delivering the bone morphogenetic protein 2. This scaffold is used to repair a sheep metatarsal 25‐mm long critical‐size bone defect. Results show that scaffold internal geometry, notably pore shape, influences bone regeneration. Cubic geometry induces the best bone regeneration.

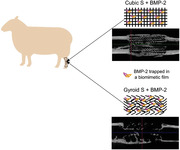

## 1 Introduction

Critical‐size bone defects, which result from high energy traumas, non‐unions, tumor resection, infection, and so forth are unable to heal by themselves.^[^ ^1^ ^]^ Thus, their reconstruction remains a challenge for clinicians and has a high economic and societal cost.^[^ ^2^ ^]^ Currently, autologous bone graft is the gold standard treatment for such defects. However, the amount of bone is limited, it induces morbidity at the donor site and the number of surgeries is globally increased. Last but not least, it leads to inconsistency in the repair of large bone defects (>5 cm^3^).^[^ ^3^, ^4^ ^]^ To tackle these drawbacks, synthetic scaffolds, made of a large variety of materials like ceramics, metals, and polymers, have been developed and used as bone graft substitutes.^[^ ^5^, ^6^, ^7^ ^]^ The induction of bone regeneration can be achieved by optimizing the properties of the scaffolds, or by including additional functionalities, notably active surface coatings,^[^ ^8^, ^9^, ^10^, ^11^, ^12^, ^13^ ^]^ delivery of osteoinductive growth factors like bone morphogenetic proteins (BMPs),^[^ ^14^, ^15^, ^16^, ^17^, ^18^, ^19^ ^]^ or stem cells.^[^ ^15^, ^20^, ^21^ ^]^ Such strategies are currently being developed in view of clinical applications.^[^ ^22^ ^]^

Growth factor‐ or drug‐based products, that is, without cells, need to follow the regulatory path for medicinal products, pharmaceutical products, or combined medical devices, depending on their intended use and whether the scaffold itself is responsible or not of the main action.^[^ ^22^, ^23^, ^24^ ^]^ BMP‐2 and BMP‐7 are the most widely used osteoinductive growth factors for bone regeneration. Combined with a collagen sponge or paste, BMPs are authorized for clinical usage in several indications, including treatment of long bones open fractures, spinal fusion, and non‐unions.^[^ ^25^, ^26^ ^]^ Unfortunately, their use became controversial due to the use of supra‐physiological doses (several milligrams) and observation of side effects in humans, including inflammation, osteolysis, bone cyst, and ectopic bone formation.^[^ ^27^ ^]^ More recently, new forms of BMP proteins have been developed, like the BV‐265 chimera, and new materials have been engineered to optimize and better localize the delivery of BMPs.^[^ ^28^ ^]^ Two major strategies are used to optimize BMP delivery in space and time. First, BMPs can be delivered in the bulk of a hydrogel or paste. In this case, BMPs are usually pre‐mixed with the hydrogel or paste, which is then either directly injected into the bone defect, or used to fill a synthetic scaffold that has sufficient properties to sustain mechanical forces. The combined product (scaffold + paste) is inserted into the bone defect. This was done with BMP‐7 loaded into a collagen carrier, which was inserted in the inner duct of a composite synthetic scaffold made of polycaprolactone (PCL) and β‐tricalcium phosphate (β‐TCP).^[^ ^15^, ^29^ ^]^ The second strategy is to incorporate BMP into a surface coating,^[^ ^30^, ^31^ ^]^ which is itself deposited at the surface of a synthetic scaffold. By doing so, the properties of the scaffold (mechanical, physical, and internal architecture) are controlled independently of the bioactivity of the BMP protein. In addition, the BMP is delivered by the surface coating at the scaffold surface, which enables to localize and control spatially its delivery.

There is a broad range of possibilities to graft or adsorb BMP at surfaces. Interestingly, layer‐by‐layer films of polyelectrolytes were shown to be particularly adapted for loading controlled doses of BMP‐2 over large ranges, and subsequently releasing it in vivo to induce bone repair. Indeed, the film properties, including composition, thickness, biodegradability, crosslinking, and BMP‐2 loading can be controlled at an implant surface.^[^ ^32^, ^33^ ^]^ We previously evidenced that a film based on hyaluronic acid (HA) and poly(l‐lysine) (PLL) can deliver tunable doses of BMP‐2 to repair a critical‐size bone defect in a rat femur.^[^ ^34^ ^]^ Such films could also withstand sustained drying and sterilization by γ‐irradiation, while still being osteoinductive in an ectopic site.^[^ ^35^ ^]^ Advantageously, recent developments in additive manufacturing techniques enable to fabricate scaffolds of any size, shape, internal architecture, and porosity.^[^ ^7^, ^36^ ^]^ Very recently, we used an additive manufacturing technique, fused deposition modeling (FDM),^[^ ^36^ ^]^ to fabricate architectured polymeric scaffolds made of polylactic acid (PLA). These scaffolds were used to repair a critical‐size bone defect in a 12 cm^3^ volumetric bone defect in the minipig mandible.^[^ ^37^ ^]^ Bone repair was found to be efficient and to depend on the BMP‐2 dose delivered via the polyelectrolyte film.

In the present study, we aimed at optimizing the repair of a critical‐size defect in sheep metatarsal bone that is representative of human long bones such as tibia or femur.^[^ ^38^ ^]^ This defect was treated with a 3D‐printed architectured scaffold in combination with an osteoinductive coating. Our main goal was to optimize bone repair by modulating the internal architecture and porosity of the synthetic polymeric scaffold. Our secondary goal was to assess the biocompatibility and biodegradability of the film‐coated scaffold in vitro on cells, and in vivo in a small animal model, according to regulatory standards for implantable biomaterials, following NF EN ISO 10 993 guidelines.

First, we designed a scaffold to repair a 25‐mm long metatarsal bone defect in sheep by modulating its internal architecture (pore shape and pore size). The scaffolds made of PLA were 3D‐printed using a FDM 3D printer. In a second step, they were coated with an osteoinductive PLL/HA film, and finally, they were loaded with a controlled dose of BMP‐2. Biocompatibility and biodegradability tests were performed on customized PLA discs, the type of disc being adapted to in vitro assays on cells and in vivo experiments in rats. The assays were done according to the regulatory standards for implantable biomaterials. The efficiency of the 3D coated scaffolds to repair a sheep metatarsal bone defect was assessed for 4 months. Together, our results showed that the synthetic polymeric scaffolds with cubic pores of ≈870 µm, coated with a biomimetic film loaded with a controlled BMP‐2 dose, can repair efficiently a sheep metatarsal critical‐size bone defect. To our knowledge, this is the first time a synthetic bone graft made of a 3D scaffold and a bioactive film is used to repair a long bone defect in a large animal (sheep). It is also the first time that the influence of scaffold geometry is studied in such a critical‐size bone defect. This opens perspectives for a future clinical application of such scaffolds that can be personalized to each patient.

## 2 Results

### 2.1 Design of 2D and 3D PLA Scaffolds for In Vitro and In Vivo Experiments

**Figure 1:**
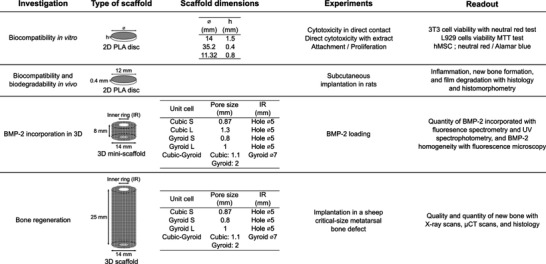
Summary of the experimental design. Each investigation is presented: Type of scaffold used, dimension of the scaffold, type of experiments and readout of the experiments.

In order to assess all aspects of the newly developed synthetic bone grafts from their in vitro biocompatibility to their efficiency to repair a critical‐size bone defect in a large animal, the study was divided in four complementary parts (**Figure** **1**): i) in vitro biocompatibility studies, notably cytotoxicity and stem cell response, on either film‐coated or uncoated 2D PLA discs; ii) in vivo biocompatibility and biodegradability evaluation of 2D PLA discs in rat as small animal model; iii) assessment of the influence of scaffold geometry on BMP‐2 loading in 3D mini‐scaffolds; and iv) bone healing of critical‐size metatarsal bone defect in sheep using BMP‐2‐containing 3D scaffolds of different geometries. Figure 1 summarizes all experiments conducted and provides information on the custom‐designed scaffolds, including their dimensions, experiment type, and qualitative and quantitative readouts.

First, in vitro biocompatibility assays were performed to ensure that PLA coated with the biomimetic film was not cytotoxic and favored a normal behavior of stem cells. 2D discs of different sizes that could be inserted in different types of multiwell cell culture plates were fabricated to perform cellular assays according to NF EN ISO 10 993 guidelines. Similar 2D discs with slightly different dimensions were used for subcutaneous implantations in rats to evaluate biocompatibility and biodegradability in vivo (Figure 1).

The external geometry of 3D porous scaffolds fabricated for the sheep study was designed based on the bone shape resected during osteotomy, that is, a tube (cortical bone) with a central empty core (medullary cavity) (Figure S1, Supporting Information). Therefore, cylinders of 25 mm in height and 14 mm in diameter with a differentiated inner ring were designed as external shape of the 3D scaffolds (Figure 1). Three types of geometric repeating unit cells (cubic, gyroid, and cubic‐gyroid, a combination of cubic and gyroid) were selected for the inner structures according to the analysis of the literature. Designs of experiments (DOE) were then performed to determine pore sizes as a function of the unit cell shapes. Three geometries were initially selected (Figure 1): i) Cubic S made of a thick hollow cylinder with opened cubic pores of ≈0.87 mm and with a hollow inner ring of 5 mm in diameter. According to the predictive values obtained by the DOE, its mechanical properties were lower than those of sheep native bone, that is, a compressive modulus of ≈300 MPa versus 28 GPa for native cortical bone and compressive strength of ≈7 MPa versus 16.6 MPa for native trabecular bone (no data was found for native cortical bone).^[^ ^39^, ^40^ ^]^ ii) Gyroid L made of a thick hollow cylinder with ≈1 mm mean pore size and a hollow inner ring of 5 mm in diameter. Such structure was selected for its triply‐periodic minimal surface design that is characterized by a zero mean curvature enhancing the surface area to volume ratio and that mimics trabecular bone structure.^[^ ^41^ ^]^ According to the DOE, its compressive modulus was ≈100 MPa and its compressive strength ≈3 MPa. iii) Cubic‐Gyroid made of an outer thick cylinder with ≈1.1 mm cubic opened pores and an inner ring of 7 mm in diameter filled with ≈2 mm gyroid pores. This latter geometry was selected as it combined the features of both cubic and gyroid structures, and we hypothesized that such composite structure may impact bone regeneration. According to the DOE, its compressive modulus and strength were respectively of ≈220 MPa and ≈6 MPa. Following the preliminary experiment in sheep, a fourth geometry was evaluated: iv) Gyroid S made of a thick hollow cylinder with ≈0.81 mm pores and a hollow inner ring of 5 mm in diameter. According to the DOE, its compressive modulus was ≈120 MPa and its compressive strength was ≈3.5 MPa. The porosity of all selected geometries was >75% according to the DOE.

In order to assess the impact of pore size and pore shape on the BMP‐2 loading within 3D architectured scaffolds, 3D mini‐scaffolds were specifically designed and printed (Figure 1). A geometry that was not considered for implantation was used in this BMP‐2 incorporation study: Cubic L, a cubic geometry with pores of ≈1.3 mm. This geometry was used in the BMP‐2 loading experiments since it had the same pore shape as Cubic S and the same surface as Gyroid L. Thus, it allowed to study the effect of pore size for two different pore shapes (cubic and gyroid) and pore shape for two different pore sizes (small (S) or large (L)) on BMP‐2 loading. Cubic L was not considered for implantation in sheep metatarsal bone defect because it had too large pores, making it mechanically too brittle to be manipulated by surgeons.

It is worth noting that, in view of future clinical studies, clinical‐grade (cgPLA) PLA was used for the fabrication of scaffolds tested in vivo, while in vitro experiments, except BMP‐2 incorporation in 3D, were performed using regular‐grade PLA (rgPLA) for financial reasons. Indeed, cgPLA costs ≈1000 times more than rgPLA. 2D discs and 3D scaffolds were designed and 3D‐printed for the specific purpose of each experiment.

### 2.2 PLA Scaffolds Coated with a Bioactive Film are Biocompatible In vitro

**Figure 2:**
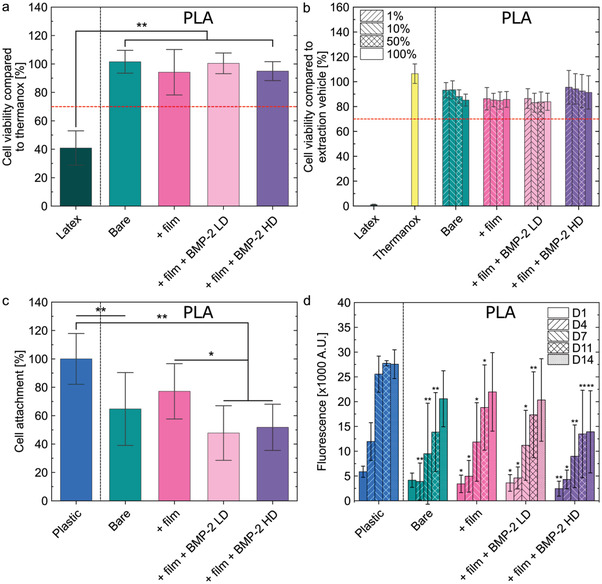
In vitro biocompatibility assays on 2D PLA discs. a) Direct cytotoxicity with contact. Cell viability compared to thermanox (expressed in %) is shown for each experimental condition: latex (positive control), bare PLA discs, film‐coated PLA discs, and film‐coated PLA discs loaded with two doses of BMP‐2: LD and HD. ANOVA with Bonferroni post‐hoc analysis was used. b) Direct cytotoxicity with extract. Cell viability compared to an extraction vehicle (expressed in %) is shown for each experimental condition. c) Cell attachment compared to plastic (expressed in %) for each experimental condition. Kruskal–Wallis ANOVA with Dunn's test was used. d) Cell proliferation, expressed as fluorescence arbitrary unit, for each experimental condition. Conditions were compared to the plastic control. Kruskal–Wallis ANOVA with Dunn's test was used. In all panels of this figure, experiments were performed with *n* = 3 or 4 samples per condition in each independent experiment. Data are represented as mean ± SD. *p < 0.05; **p < 0.01.

For direct cytotoxicity assay (**Figure** **2**a), a material substrate is considered toxic if it promotes a reduction of 3T3 fibroblast viability by more than 30% compared to the one on negative control Thermanox substrate (set at 100%). Whereas cell viability was 41% on the positive control latex substrate, it remained higher than 94% on all PLA substrates tested, indicating that none of the biomaterial substrates induced cytotoxicity (Figure 2a). In addition, the viability of L929 cells in the presence of extracts from each of the 2D disc tested was always higher than 83% (Figure 2b), confirming the absence of cytotoxicity of all biomaterials tested.

The analysis of human mesenchymal stem cells (hMSC) attachment to biomaterial substrates after 15 h of culture showed that, compared to control plastic, ≈35% fewer cells attached onto the PLA surface in the absence or presence of the biomimetic film (Figure 2c). The presence of BMP‐2 in the film further decreased cell attachment by ≈27%, independently of the BMP‐2 dose. Additionally, the proliferative rate of hMSC was lower on PLA compared to control plastic substrate (Figure 2d). On plastic, cell number reached a plateau after around 7 days of culture, whereas no plateau was reached on bare PLA after 14 days of culture. However, a plateau was nearly reached on film‐coated PLA with and without BMP‐2 LD after 14 days, and a plateau was reached at 11 days on film‐coated PLA loaded with BMP‐2 HD. While coating PLA with the biomimetic film did not affect cell proliferation, the addition of BMP‐2 decreased cell proliferation. This decrease was more marked with the highest BMP‐2 dose. This may be explained by the fact that BMP‐2 induced the osteoblastic differentiation of hMSC, thus blocking their proliferation. Differentiation tests should be conducted to assess this hypothesis.

Together, these results showed that 3D‐printed PLA coated with the biomimetic film with or without BMP‐2 was not cytotoxic. Furthermore, adding BMP‐2 in the film reduced cell attachment but not in a dose‐dependent way and increasing the BMP‐2 dose decreased cell proliferation.

### 2.3 In Vivo Biocompatibility and Biodegradability of Film‐Coated PLA Discs in Small Animals

To assess the biocompatibility and kinetics of resorption of film‐coated PLA discs, 36 adult Wistar rats have been subcutaneously implanted with four samples each of bare PLA, film‐coated PLA without BMP‐2, or film‐coated PLA with BMP‐2 loaded at two different BMP‐2 concentrations: BMP‐2 LD for low dose and BMP‐2 HD for high dose (Figure S2a, Supporting Information). The clinical and weight follow‐up lasted 7, 28, or 48 days (*n* = 3 rats per time point, i.e., 12 samples per condition). Then, animals were euthanized and vital organs (liver, spleen, heart, brain, and kidney) and implanted tissue was explanted for histological analysis.

The clinical recovery was normal and healing of the skin evolved normally over time whatever the condition. The implant was always retrieved inserted into the skin, integrated into a fibrous capsule (Figure S2b, Supporting Information). No infection occurred.

**Figure 3:**
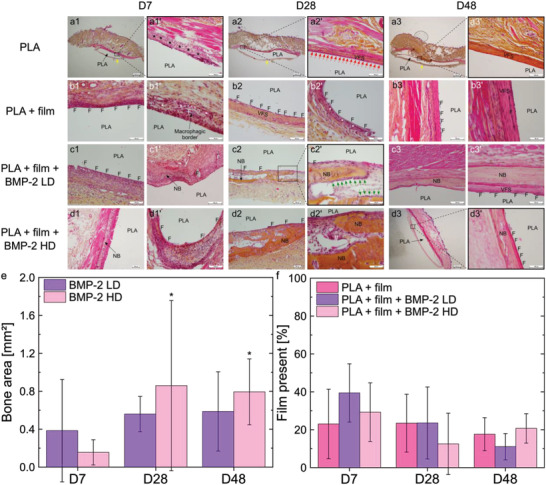
In vivo biocompatibility and biodegradability of films in rats quantified over time. Images of sections of PLA discs taken as a function of time at D7, D28, and D48 (endpoint) for the four experimental conditions studied: PLA; PLA + film; PLA+ film + BMP‐2 LD; PLA + film + BMP‐2 HD. a1–a3,d3) Scale bar is 1 mm. a1’, b1,c1–d1’,a2’,b2,c2,d2,a3’,b3,c3,d3,d3’) Scale bar is 100 µm. b1’,b2’,c2’,d2’,b3’c3’) Scale bar is 50 µm. Yellow arrows: fibrous capsule. (*) Macrophages and/or polynuclear cells. F: biomimetic film. NB: new bone. Red arrows: macrophagic border. VFS: vascularized fibroblastic shell. Green arrows: osteoblasts. (#) Giant multinucleated cells. e) Quantification of the bone area (mm^2^) formed at D28 and D48 when BMP‐2 was used. ANOVA with Bonferroni post‐hoc analysis was used. * *p* < 0.05 compared to D7. f) Quantitative analysis of the amount of film (%) remaining on the biomaterials surface. Kruskal–Wallis ANOVA with Dunn's test was used and showed that there was no significant statistical difference between conditions. In (e,f), for each time point, there were *n* = 12 samples per condition using *n* = 3 rats, each receiving four implants. Data are represented as mean ± SD.

Histological analysis of the different vital organs did not reveal any histological abnormality (data not shown). Moreover, it was shown that the PLA discs never resorbed but were often not adherent to the surrounding tissue, with a fibrous capsule (yellow arrows) around them (**Figure** **3**a,e,i). Time points are hereafter noted DX with X being the number of days after implanation. A macrophagic inflammatory reaction with polynuclear cells (*) around the 2D PLA discs was observed at D7 (Figure 3a′), as expected for a normal reaction to a foreign body. This reaction decreased at D48 compared to D28 (Figure 3e–l′). The film (F) remained visible for all substrates tested at all‐time points (Figure 3b,f,g,h,l′). However, it was sometimes peeled off from the PLA discs (Figure 3c,d′,j,k′) and some fragments were totally detached and surrounded by a macrophagic border with polynuclear cells (Figure 3b′) or giant multinucleated cells (#) (Figure 3h′), or they were encapsulated into a fibrous capsule (Figure 3c′) or in a vascularized fibroblastic shell (VFS) (Figure 3j′). Sometimes, macrophages were located next to the film (Figure 3f′). At D28 and D48, a vascularized fibroblastic shell in the periphery of the implant was observed (Figure 3e′,f,i′,j,k′). This fibroblastic shell was delimited by a macrophagic border (red arrows) at D28 (Figure 3e′). No bone formation was observable for bare PLA and film‐coated PLA without BMP‐2 at any time points (Figure 3a,b′,e,f′,i,j′). At D7, some new bone (NB) was forming in contact with the biomaterial with BMP‐2 LD (Figure 3c′) and new bone was visible with BMP‐2 HD (Figure 3d). At D28 and D48, new bone formation was observed for both BMP‐2 doses (Figure 3g,h′,k,l′), with sometimes bone tissue surrounded by osteoblasts (green arrows) (Figure 3g′). Histomorphometry showed that at D7, PLA + film + BMP‐2 LD formed ≈1.5 times more bone compared to PLA + film + BMP‐2 HD. On the contrary, at D28, 54% more bone was formed with BMP‐2 HD compared to BMP‐2 LD and at D48, 35% more bone was formed with BMP‐2 HD compared to BMP‐2 LD. The quantity of new bone formed with BMP‐2 LD was not significantly higher at D28 and D48 compared to D7 because of high variability at D7. On the contrary, significantly more bone was formed at D28 and D48 compared to D7 when using BMP‐2 HD. Interestingly, the quantity of newly formed bone did not increase between D28 and D48 (Figure 3m). A two‐way ANOVA test was performed and showed that there was no significant difference in the quantity of new bone formed between BMP‐2 LD and BMP‐2 HD, but there was a significant difference between the quantity of bone formed at D7 and the quantity of bone formed at D28 and D48. The film present at the implant surface at each time point of the experiment was also evaluated: significantly more film was found to be present at D7 compared to D28 and D48, with however no significant difference between the experimental conditions.

### 2.4 BMP‐2 Incorporation in 3D Scaffolds

The study of BMP‐2 loading in the different 3D architectures was conducted in vitro in 3D mini‐scaffolds made of cgPLA.

**Figure 4:**
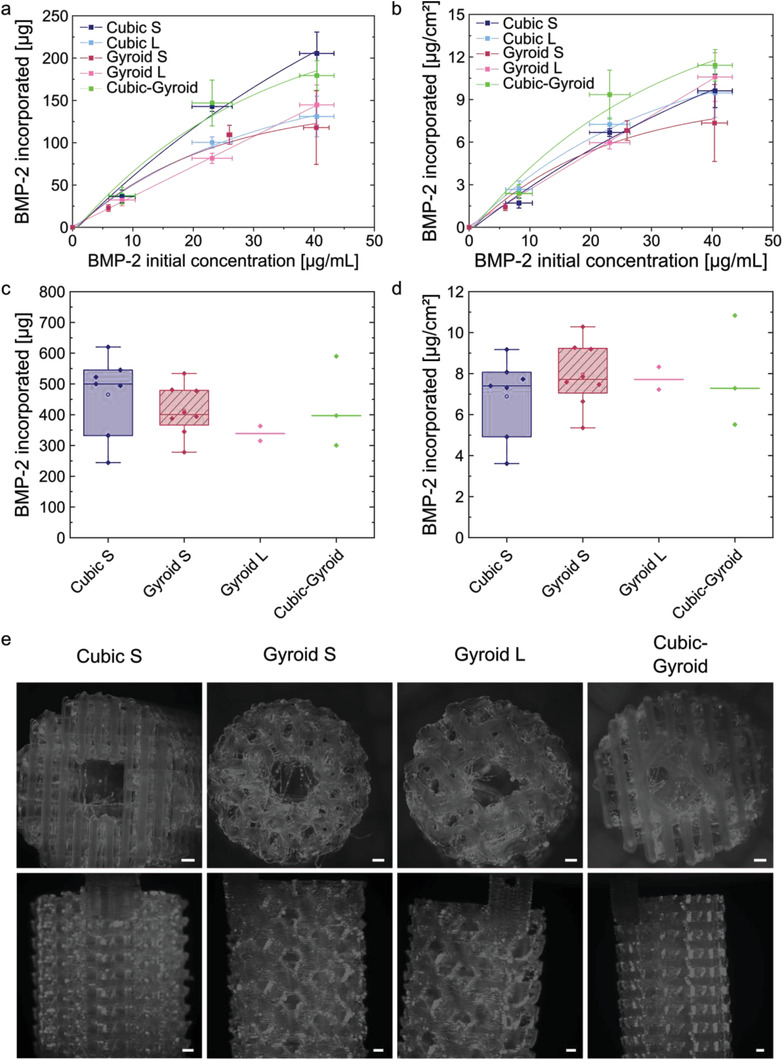
Characterization of BMP‐2 loading in 3D mini‐scaffolds. a) Quantification of the total dose of BMP‐2 incorporated in 3D mini‐scaffolds as a function of the BMP‐2 initial concentration in the loading solution expressed as absolute mass (µg) and b) expressed as surface dose in µg cm^−^^2^. The parameters extracted from the fits of the data are given in Table S2a,b, Supporting Information, respectively. *n =* 3 scaffolds per geometry. Data are expressed as mean ± SD. c) Total dose of BMP‐2 incorporated in 3D scaffolds prepared for in vivo experiments in sheep (µg) as a function of scaffold geometry. d) BMP‐2 dose incorporated in 3D scaffolds prepared for in vivo experiments in sheep expressed as surface dose (µg cm^−^^2^) after normalization by the scaffold effective surface, as a function of scaffold geometry. In (c,d), *n =* 7 for Cubic S, *n =* 8 for Gyroid S (one scaffold was not implanted), and *n =* 2 for Gyroid L and Cubic‐Gyroid. Data are presented by median and interquartile range. ANOVA with Bonferroni test was used and showed that there was no significant statistical difference between conditions. e) Fluorescence macroscopy images of 3D scaffolds loaded with BMP‐2^Rhod^ for each studied geometry Cubic S, Gyroid S, Gyroid L, and Cubic‐Gyroid. Scale bar is 1 mm.

Micro‐compted tomography (µCT) images of the different 3D mini‐scaffolds, along with their surface, porosity, and pore size, are shown in Figure S3, Supporting Information. The total amount of BMP‐2 incorporated (in µg) is given for each type of geometry, as a function of BMP‐2 concentration in the loading solution (**Figure** **4**a). The notation BMPXX will be further used to design BMP‐2 at a concentration of XX µg mL^−1^ in the loading solution. Effective surface doses incorporated (µg cm^−^^2^) are also given to take into account the differences in scaffold surfaces (Figure 4b and Figure S4, Supporting Information). The % of BMP‐2 from the initial loading solution effectively loaded in the scaffolds is also given (Figure S5, Supporting Information). The incorporation of BMP‐2 increased with the increase of BMP‐2 concentration in the loading solution (Figure 4a,b). However, differences appeared between geometries: BMP‐2 incorporation in Gyroid L appeared to increase linearly while it was not linear for the other geometries (Table S2, Supporting Information). Moreover, BMP‐2 loading in Gyroid S reached a plateau, while this was not the case for Cubic S and Cubic‐Gyroid (Figure 4a,b). Figure S4, Supporting Information, shows that, at BMP10, Cubic L and Gyroid L incorporated significantly more BMP‐2 than Cubic S and Gyroid S, respectively, indicating that pore size influenced BMP‐2 loading at low BMP‐2 dose. At BMP30, Cubic L incorporated significantly more BMP‐2 than Gyroid L, suggesting that pore shape influenced BMP‐2 loading. At BMP50, Cubic S incorporated significantly more BMP‐2 than Gyroid S, again suggesting an effect of pore shape on BMP‐2 loading. Moreover, the BMP‐2 incorporation in Gyroid S and Gyroid L were significantly different at BMP30 and BMP50. These results showed that pore size and shape, so scaffold geometry, influenced BMP‐2 incorporation.

For the in vivo implantation in a sheep metatarsal critical‐size bone defect, a dose of 500 µg of BMP‐2 per scaffold was targeted. As the scaffolds had different geometries, their surfaces were different and thus the concentration of BMP‐2 in the loading solution was adapted to each scaffold geometry. The doses of BMP‐2 incorporated into large 3D scaffolds used for in vivo implantation in sheep are shown in Figure 4c,d. There were some differences in BMP‐2 incorporation between all geometries, but not statistically significant (Figure 4c): BMP‐2 loading was the highest in Cubic S and the lowest in Gyroid L scaffolds. In terms of effective BMP‐2 surface doses, similar BMP‐2 doses were loaded in all geometries (Figure 4d).

Finally, the homogeneity of BMP‐2 loading inside the 3D scaffolds was visualized by fluorescence macroscopy and microscopy using BMP‐2 labeled with 5(6)‐Carboxytetramethylrhodamine N‐succinimidyl ester (BMP‐2^Rhod^) (Figure 4e, details in Supporting Information, and Figure S6, Supporting Information). BMP‐2 appeared to be homogeneously distributed within the 3D scaffolds. All together, these data showed that BMP‐2 was efficiently loaded onto the surface of 3D porous scaffolds of various architectures designed for in vivo implantations, and that the incorporated BMP‐2 dose was similar in all scaffolds.

The amount of BMP‐2 released from the biomimetic film as a function of the BMP‐2 initial concentration in solution and of the film crosslinking level, as well as in function of time, has already been quantified in vitro.^[^ ^34^, ^37^ ^]^ In the in vivo study in sheep, the amount of BMP‐2 released in the bloodstream was quantified by Elisa assay after collecting sera from sheep at early time points following implantation (0, 1, 2, 4, and 7 days). It was found undetectable (data not shown).

### 2.5 Physico‐Chemical, Mechanical, and Morphological Characterization of 3D PLA Scaffolds and Film Coating Prepared for In Vivo Implantation in Sheep

**Figure 5:**
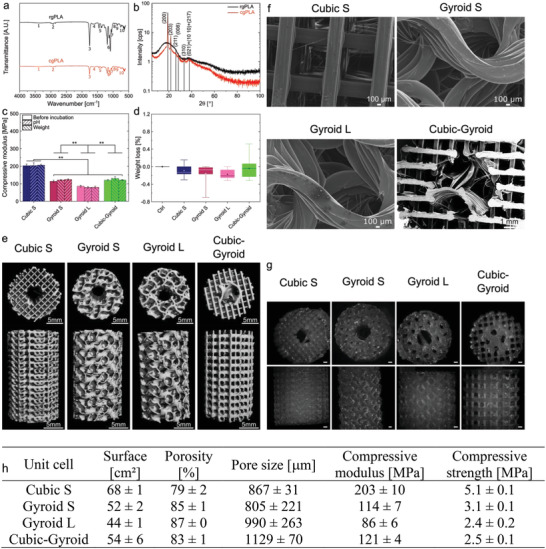
Physico‐chemical, mechanical, and morphological characterization of PLA scaffolds and film coating. a) ATR‐FTIR transmittance spectra of rgPLA and cgPLA. Remarkable peaks are numbered and identified in Table S3, Supporting Information. b) SAXS spectra of rgPLA and cgPLA. Remarkable peaks are identified with Miller indices on the graph and in Table S4, Supporting Information. c) Compressive modulus (MPa) of the different scaffold geometries measured at different time points of the experiment: before incubation, after the immersion in a physiological solution over 12 weeks (scaffolds were never dried), and after the weight loss experiment, for which scaffolds were dried at each time point before weighing. Data are presented as mean ± SD. **p < 0.01. d) Weight loss (expressed in %) measured for the scaffolds of different the geometries Cubic S, Gyroid S, Gyroid L, and Cubic‐Gyroid. Data are presented by median and interquartile range. In (c,d), *n =* 3 for each condition and ANOVA with Bonferroni test was used. e) µCT scans of the different scaffold geometries. f) Representative SEM images of the top surface of the different scaffolds for each geometry. g) Fluorescence macroscopy images of PLL^FITC^ coated‐scaffolds (scale bar: 1 mm). h) Table recapitulating all measured values for each geometry (*n* = 3): effective surface (in cm^2^), porosity (in %), pore size (in µm), compressive modulus (in MPa), compressive strength (MPa).

Characterization of the different 3D scaffold geometries was performed using complementary techniques (**Figure** **5**). First, the structural differences between the two types of PLA filaments, rgPLA and cgPLA, were analyzed using attenuated total reflectance Fourier transform infrared spectroscopy (ATR‐FTIR) (Figure 5a) and small angles X‐ray scattering (SAXS) (Figure 5b) to compare their chemical composition and crystallinity. ATR‐FTIR showed no difference between the end‐groups of both polymer chains, indicating that the filament structures were similar (Figure 5a and Table S3, Supporting Information). Moreover, two peak ratios of interest, *R*_1_ (1209/1180 cm^−1^ band intensity) and *R*_2_ (1130/1080 cm^−1^ band intensity) indicate the degree of PLA crystallinity (the higher these ratios are, the more crystallized PLA is).^[^ ^42^ ^]^ Higher values were found for cgPLA (*R*_1_ = 0.62 and *R*_2_ = 0.67) compared to rgPLA (*R*_1_ = 0.54 and *R*_2_ = 0.52), suggesting that cgPLA was more crystallized than rgPLA (Figure 5a). Regarding SAXS, six diffraction peaks were identified for cgPLA compared to the three peaks identified for rgPLA, confirming the higher degree of crystallinity of cgPLA compared to rgPLA (Figure 5b and Table S4, Supporting Information).

The in vitro degradation of the 3D PLA scaffolds was assessed by measuring the pH variation of the incubating solution (an indicator of the possible release of acidic products, Figure S7a,b, Supporting Information), the scaffold weight loss (Figure 5c; Figure S7c, Supporting Information), and the scaffold mechanical properties (Figure 5d; Figure S7d, Supporting Information), which were measured in physiological conditions in a phosphate buffered saline solution (PBS) as a function of time (details in Supporting Information, Figure S7, Supporting Information). The pH variation was the highest during the two first weeks of the incubation time and then decreased, but remained overall <4% for all scaffolds (Figure S7a, Supporting Information). Gyroid L exhibited the lowest pH variation (Figure S7b, Supporting Information). Cubic S with and without film coating, Gyroid S, and Cubic‐Gyroid with film coating were different from the negative control, for example, pure PBS without any scaffold. According to a two‐way ANOVA statistical test with one factor being the geometry and the other one being the presence of the film, pH variation for Cubic S was statistically different from pH variation for Gyroid L, meaning that more acidic products were released by Cubic S (Figure S7b, Supporting Information). The presence of the film on the scaffolds slightly increased pH variations for all tested geometries, indicating that more acidic products were released, although with minor changes (no statistical difference). The weight loss remained <1% over the incubation time, and was negative during the two first weeks, presumably due to water uptake by PLA fibers. Then, it progressively increased and was the lowest for Gyroid L (Figure 5c; Figure S7c, Supporting Information). Mechanical properties of the 3D PLA scaffolds were assessed using uniaxial compressive tests (Figure 5d; Figure S7d, Supporting Information). The compressive modulus and strength had lower values than those predicted by the DOE. Incubation in PBS during 12 weeks did not have a significant impact on scaffold mechanical properties as can be seen by the constant values of the compressive modulus and strength (Figure 5d; Figure S7d, Supporting Information).

The 3D PLA scaffolds with different geometries were visualized with µCT scans (Figure 5e). Quantitative parameters were deduced from the µCT scans, including their effective total surface area (in µm^2^), porosity (given in %), and mechanical properties, which are summarized in Figure 5h. Cubic S and Gyroid L geometries displayed the highest (68 cm^2^) and the lowest (44 cm^2^) surfaces, respectively. Porosity values ranged from 79% to 87%, which should be sufficient to enable cell invasion and vascularization.^[^ ^43^ ^]^ Scanning electron microscopy (SEM) was used to quantify pore sizes (Figure 5f,h), which were found to vary from 805 to 1130 µm. Cubic‐Gyroid had the largest pores of ≈1130 µm and Gyroid S the lowest of ≈805 µm. Regarding the mechanical properties, the compressive modulus was in the range from ≈85 to 200 MPa and compressive strength from ≈2.5 to 5 MPa (Figure 5h; Figure S8, Supporting Information). Cubic S had the highest compressive modulus (203 MPa) and compressive strength (5.1 MPa), while Gyroid L had the lowest (86 and 2.4 MPa respectively).

The homogeneity of the film coating onto the PLA fibers of porous 3D scaffolds was assessed by fluorescence macroscopy using PLL labeled with fluorescein 5‐isiothiocyanate (PLL^FITC^) as last layer of the coating (Figure 5g).^[^ ^44^ ^]^ Film coating inside the bulk of the 3D scaffolds was also assessed after cutting them into pieces and imaging the slices. All geometries were fully and homogeneously coated by the film (details in Supporting Information and Figure S9, Supporting Information). Film thickness in dry state was estimated to be ≈2 µm based on SEM imaging (details in Supporting Information and Figure S10, Supporting Information).

### 2.6 Preliminary Experiment to Assess the Influence of Scaffold Geometry on Bone Regeneration

A preliminary experiment with a reduced sample size (*n* = 2 per geometry) was conducted to select the optimal scaffold geometries (Cubic S, Gyroid L, or Cubic‐Gyroid scaffolds). Scaffolds were implanted in 6 Pré‐Alpes sheep, with one scaffold per sheep (Figure S11, Supporting Information). Animals remained in good health. There was neither postoperative infection, nor implant failure. During explantation, it was impossible to distinguish macroscopically the newly formed bone from native bone or scar tissue around the implant.

**Figure 6:**
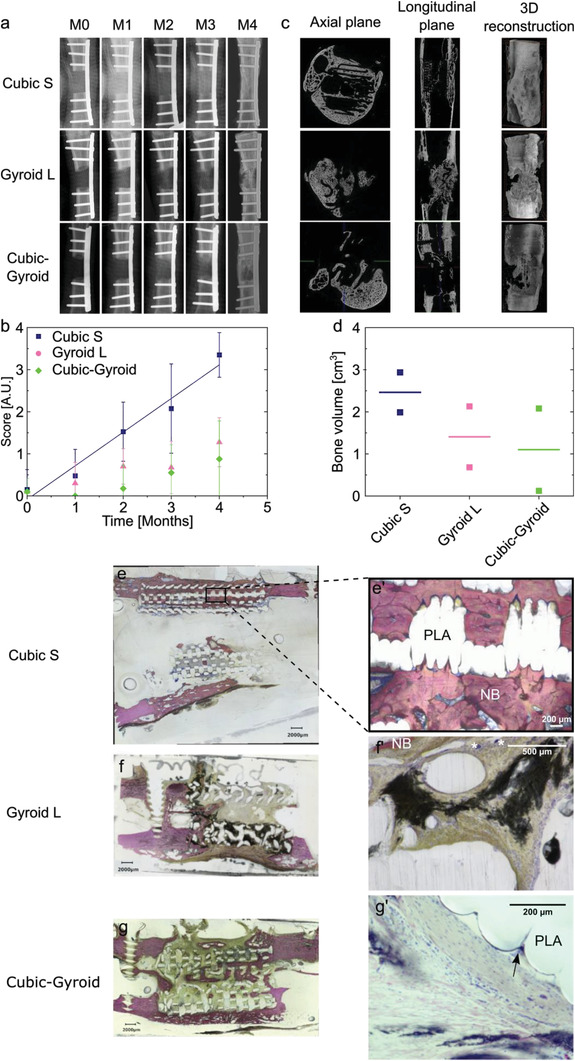
Preliminary experiment in sheep metatarsal critical‐size bone defect to assess the influence of scaffold geometry on bone regeneration. a) Representative X‐ray scans of bone regeneration achieved with film‐coated scaffolds loaded with BMP‐2 with different internal geometries at different time points: right after scaffold implantation (M0), after one, two, three months (M1, M2, M3), and after explantation (M4). b) X‐ray score given by the clinicians as a function of time for each scaffold geometry. Data are represented as mean ± SD of scores given by five clinicians and veterinarians. The scores for Cubic S were linearly fitted (*R* ^2^ = 0.97). c) Representative µCT scans acquired after explantation for each scaffold geometry. For each geometry, scans in the axial plane, longitudinal plane, and a 3D reconstruction are shown. d) Quantification of the newly formed bone volume for each scaffold geometry (*n =* 2 implants per geometry). e–g’) Representative histological sections for each scaffold geometry: Cubic S, Gyroid L, and Cubic‐Gyroid. For each geometry, a global section and a magnified view are given. Bone is stained in pink. NB: new bone. (*) Giant cells. Black arrow: multinucleated giant cells.

The radiolucent property of the scaffold PLA material facilitated longitudinal X‐rays analysis of the metatarsal bone defect. Time‐dependent increase of the radiopacity throughout the defect was observed in both animals implanted with Cubic S, indicating an early and progressive bone formation in this specific scaffold. In contrast, a limited bone formation confined to the vicinity of the edges of the defect filled with either Gyroid L or Cubic‐Gyroid scaffolds was observed up to 3 months post‐implantation leading to a partial bone formation in these implants at 4 months (**Figure** **6**a). One animal implanted with Cubic‐Gyroid did not form bone at all (data not shown). These observations were confirmed by the X‐ray scores, which increased over time and were the highest for Cubic S. (Figure 6b; Figure S12, Supporting Information).

µCT scans showed that the new bone grew in Cubic S primarily in the continuity of the cortices leading to bone union, whereas it grew non‐uniformly in the defects filled with Gyroid L and Cubic‐Gyroid scaffolds (Figure 6c). Bone quantification confirmed that Cubic S geometry promoted higher bone formation compared to the other geometries (mean of 2.5 cm^3^, Figure 6d). Bone homogeneity was also higher with Cubic S (Figures S13 and S14, Supporting Information). Interestingly, the newly formed bone in Cubic S was similarly distributed in all areas of the defect, namely proximal, central, and distal bone while it tended to be more localized in the proximal and distal areas in Gyroid L and in the proximal area in Cubic‐Gyroid. Ectopic bone formation was also lower in Cubic S than with the other geometries (Figures S13 and S14, Supporting Information).

The histological examination indicated abundant new bone tissue inside and around the pores of Cubic S scaffolds (Figure 6e). Scaffold struts were either surrounded by newly formed bone (NB) (Figure 6e′) or by a fibrous tissue. More fibrous tissue was observed within the Gyroid L and Cubic‐Gyroid scaffolds with the presence of islets of new bone (Figure 6f,g) but a larger magnification of another section shows some inflammatory cells and giant cells (*) (Figure 6f′). Cubic‐Gyroid led to some new bone formation inside the scaffold (Figure 6g), and some multinucleated giant cells were observed (Figure 6g′, black arrow). Globally, this histological analysis confirmed scaffold geometry influenced bone regeneration and that Cubic S led to the best bone regeneration among the three geometries tested.

Based on this preliminary experiment, we decided to pursue our investigations with Cubic S, which induced the highest amount of newly formed bone, had high mechanical properties and high porosity. For the second main experiment, we decided to add a gyroid geometry with a pore size similar to Cubic S, that is, ≈805 µm, hereafter named Gyroid S. These two geometries only differ in their pore shape.

### 2.7 Main Experiment to Study the Influence of Pore Shape and Optimize Bone Regeneration

BMP‐2‐loaded Cubic S (*n* = 5, completed with the 2 sheep from the preliminary experiment to reach a sample size of *n* = 7) and Gyroid S (*n* = 7) scaffolds as well as two controls per geometry (film‐coated scaffolds without BMP‐2) were implanted in sheep. Similarly to the preliminary experiment, animals remained in good health with no postoperative problem, and the newly formed bone was well integrated.

**Figure 7:**
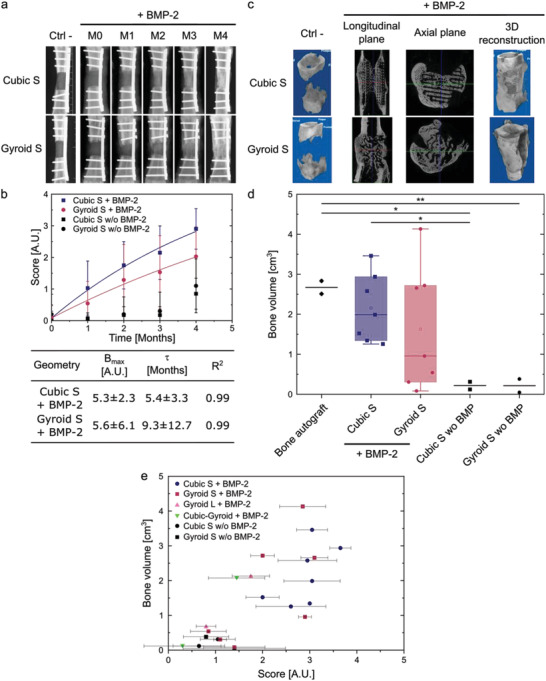
Main experiment in sheep metatarsal critical‐size bone defect to assess the influence of pore shape and optimize bone regeneration. a) Representative X‐ray scans acquired at different time points: right after scaffold implantation (M0), after one, two, three months (M1, M2, M3), and after explantation (M4) for each scaffold geometry. b) X‐ray score as a function of time (same calculation as in Figure 6). The scores for Cubic S and Gyroid S loaded with BMP‐2 were fitted with an exponential function $y=B_{\max}+A*\exp\left(-\frac{t}{\tau }\right)$. Quantitative parameters obtained by the fit (*B*_max_ and *τ*) are given in the table. Data are presented as mean ± SD. c) Representative µCT scans acquired after explantation for each scaffold geometry. For each geometry, scans in the axial plane, longitudinal plane, and a 3D reconstruction are shown. d) Quantification of the newly formed bone volume for each scaffold geometry, in comparison to bone autograft. Data are presented as box plots with median and interquartile range. Bone autograft (*n* = 2), Cubic S and Gyroid S (*n* = 7 for each) loaded with BMP‐2. Cubic S and Gyroid S without BMP‐2 (*n* = 2 for each). Student's *t*‐tests were performed. *p < 0.05; **p < 0.01. e) Correlation between the qualitative X‐ray score given by clinicians (at 4 months) and the quantitative bone volumes deduced from µCT images: for each sample, bone volume (cm^3^) is plotted versus mean X‐ray score. Data are represented as mean ± SD. Each type of scaffold has a given symbol and color.

X‐ray scans and X‐ray scores were acquired and analyzed respectively as in the preliminary experiment (**Figure** **7**a,b). Limited bone formation was formed at the vicinity of the edges of the defects in both types of scaffolds implanted without BMP‐2 (Figure 7a). Full bridging of the defect was observed in 6/7 animals implanted with Cubic S + BMP‐2 (Figure 7a), whereas 1/7 animal led to partial bridging of the defect (Figure S15a, Supporting Information). When implanted with Gyroid S + BMP‐2, full bridging of the bone defect was achieved in 3/7 animals (Figure 7a), partial bridging in 1/7 animal (Figure S15b, Supporting Information), and scarce bone formation in 3/7 animals (Figure S15c, Supporting Information). These observations were confirmed by the X‐ray scores, which steadily increased with the implantation time in animals implanted with both types of scaffolds containing BMP‐2. As expected, scaffolds loaded with BMP‐2 displayed significantly higher X‐ray scores and indexes compared to scaffolds without BMP‐2 (Figures S16 and S17, Supporting Information). The defects filled with Cubic S + BMP‐2 showed significantly higher X‐ray scores at all‐time points, except at 2 months, compared to Gyroid S + BMP‐2 (Figure 7b; Figure S16, Supporting Information). The gap between both geometries increased with time. X‐ray scores were fitted with an exponential function providing a plateau value *B*_max_ which was found to be slightly lower for Cubic S + BMP‐2 than for Gyroid S + BMP‐2 (5.3 vs 5.6). However, this plateau was reached faster for Cubic S + BMP‐2 scaffolds (τ value of 5.4 months vs 9.3 months, respectively for Gyroid) (Figure 7b). Defects implanted with Cubic S + BMP‐2 showed significantly higher bone filling and homogeneity indexes and lower ectopic bone formation index than defects with Gyroid S + BMP‐2 (Figure S17, Supporting Information).

µCT scans provided evidence that a clinical bone union (defined as ≥ 3/4 united cortices) occurred in 5/7 and 3/7 animals implanted with Cubic S + BMP‐2 and Gyroid S + BMP‐2, respectively. This observation differed from the one made from X‐ray scans for Cubic S + BMP‐2 scaffolds on which 6/7 animals led to bone union. This difference between these two observations comes from the more limited field of view provided by 2D X‐ray scans compared with 3D µCT scans. Quantitatively, Cubic S + BMP‐2 induced higher bone formation than Gyroid S + BMP‐2 (mean of 2.2 cm^3^ vs 1.6 cm^3^, Figure 7d) but the difference was not statistically significant (*p* = 0.38). Additionally, the bone volume was significantly different in Cubic S + BMP‐2 compared to Cubic S w/o BMP‐2. However, it was not different in Gyroid S + BMP‐2 compared to Gyroid S w/o BMP‐2 presumably due to the highly variable bone volume outcomes obtained with Gyroid S + BMP‐2 (Figure 7d). The volumes of bone found in the Cubic S + BMP‐2 and Gyroid S + BMP‐2 implants were lower than the volumes found in the bone autografts (Figure 7d). It should be noted that the volume of newly formed bone by the bone autografts could not be distinguished from the volume of implanted bone graft. Therefore, the volume of newly formed bone cannot be compared between groups. In the presence of BMP‐2, the newly formed bone was homogeneously distributed within the scaffold at proximal, central and distal locations, for both Cubic S and Gyroid S geometries. In striking contrast, in the absence of BMP‐2, the new bone was mainly formed in the proximal area (Figure S18, Supporting Information).

**Figure 8:**
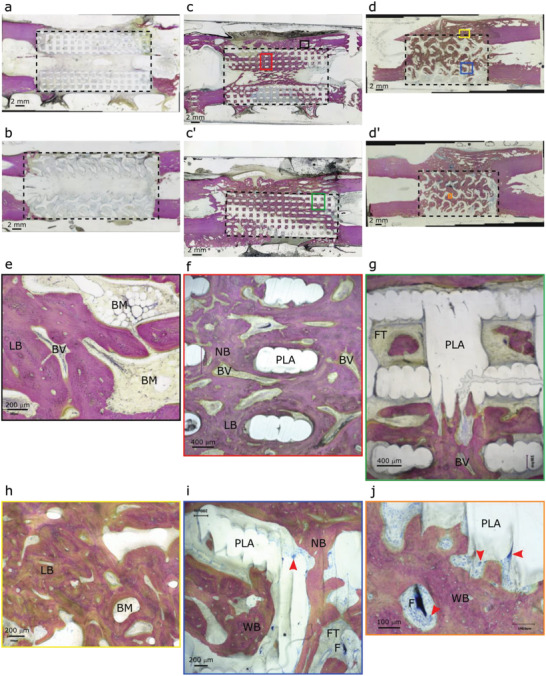
Histological analysis of scaffolds with Cubic S and Gyroid S geometries. Representative histological sections for the different scaffolds. Film‐coated PLA scaffold without BMP‐2: a) Cubic S and b) Gyroid S geometries. In the presence of BMP‐2 in the films, bone was formed in both types of scaffolds c,c’) Cubic S and d,d’) Gyroid S. At higher magnification, specific features of the newly formed bone were visible: e,f) Formation of mature lamellar bone (LB), homogeneous and dense bone with thick trabeculae filled with bone marrow (BM). Vessels (V) are also visible. g) Some remnants of the films (F) are visible, encapsulated in a fibrous capsule. h) Lamellar bone and bone marrow. i) Numerous multinucleated giant cells (red arrowheads); and j) these cells were visible close to the scaffold struts. Bone is stained in pink. PLA, PLA scaffold; BM, bone marrow; F, biomimetic film; LB, lamellar bone; NB, new bone; WB, woven bone; V, blood vessels. Scale bars: 2 mm (a–d); 400 µm (f,g); 200 µm (e,h,i); 100 µm (j).

The histological analysis of the tissue sections of defects showed that, in the absence of BMP‐2, the new bone deposition within the implant was scarce, confined to the bone ends of the defect, localized either at the periphery or in the core of the PLA scaffold, but never inside the scaffold pores ((**Figure** **8**a with Cubic S, Figure 8b with Gyroid S, and Figure S20, Supporting Information). These observations indicated that the film‐coated PLA scaffold was not osteoconductive by itself. Bone distribution inside the BMP‐2‐containing scaffolds showed some variability between animals (Figure 8c,c′,d; Figures S19 and S20, Supporting Information). In both groups, some areas of the scaffolds were filled with bone while others remained empty, providing evidence of a nonuniform bone induction. In addition, in most of the explants, the new bone tissue was mostly formed in the outer part or outside of the PLA scaffolds (peripheral area) in the continuity of the cortices, leading to a new connecting cortex when the material implant was not aligned with the native cortices (Figure 8c,c′,d; Figure S19c,c′,e,e′, Supporting Information). At higher magnification, the bone tissue formed around the scaffolds appeared mature (lamellar), homogeneous, and dense with thick trabeculae filled with bone marrow (Figure 8e,g). When found within the scaffold pores, the new bone tissue (NB) displayed either lamellar features with the presence of blood vessels (V) (Figure 8f) or woven bone features with osteocytes, bone‐lining cells and osteoblasts depositing osteoid tissue, thus, revealing an active bone formation (Figure 8i,j). However, we noted that the new bone was scarcely in contact with the PLA material struts (Figure 8g,i,j). Isolated bone nodules were also observed at a distance from the PLA struts (Figure 8g). Besides these bone nodules, infiltrated tissue including fibrous connective tissue containing blood vessels was present (Figure 8g,i,j). Numerous multinucleated giant cells (red arrowheads) were found close to or in contact with the implant PLA material (Figure 8i,j). Some remnants of the biomimetic film (F) were also observed close to the scaffold's struts, encapsulated into a fibrous capsule and most often surrounded by multinucleated giant cells (Figure 8i,j).

In summary, this main experiment provided evidence that BMP‐2 containing scaffolds consistently induced bone tissue either inside the 3D architectured scaffolds and/or at the periphery of the scaffold, while only a very limited amount of bone tissue was found in and around the scaffolds without BMP‐2. Another finding of this experiment is that Cubic S + BMP‐2 scaffolds led to a higher amount of newly formed bone when compared with Gyroid S + BMP‐2 scaffolds. The variability of the newly formed bone volumes was lower and the kinetics of bone formation was faster for Cubic S + BMP‐2 compared to Gyroid S + BMP‐2.

## 3 Discussion

In this study, we used the FDM technique to design and fabricate PLA scaffolds specifically for different in vitro and in vivo assays (Figure 1), showing the high versatility of this 3D printing technique. The characterization of PLA of clinical grade, a material required for clinical translation, revealed specific differences with PLA of regular grade, notably in terms of crystallinity. This observation highlights the need to perform the experiments with the appropriate raw material.

Regarding the material composing the scaffold, we selected PLA without any additional material in order to control independently the 3D architectured scaffold, made solely of PLA, and the 2D film coating, made of the biopolymeric film that delivers the osteoinductive protein BMP‐2. In the literature, we found an alternative strategy, like using PLA that incorporates β‐TCP particles to repair a rat femur window defect model.^[^ ^45^ ^]^ This is different in that the bioactive part is provided by the calcium phosphate. In our study, we aimed to regenerate a critical‐size bone defect in a large animal. Adding β‐TCP or even hydroxyapatite (HAP) to PLA may increase new bone formation in itself. However, HAP and β‐TCP are less osteoinductive than BMP‐2, which is to date the most potent osteoinductor. It is unsure that a PLA/HAP or PLA/β‐TCP composite scaffold (without a film coating) could repair such a critical‐size metatarsal bone defect. In addition, adding HAP or β‐TCP would render the scaffold more brittle and change its degradation rate, thus the risk of mechanical failure would be higher.

Results from the biocompatibility experiments (Figure 2) showed that hMSC adhesion was reduced on BMP‐2‐loaded films and cell adhesion was similar for BMP‐2 loaded at 30 versus 60 µg mL^−1^, indicating that a plateau was probably reached. This appears to be in contradiction with previous results obtained on C2C12 cell adhesion,^[^ ^33^ ^]^ and on human periosteum derived stem cells (hPDSCs).^[^ ^46^ ^]^ However, this effect may be due to the BMP‐2 doses used here, respectively 30 and 60 µg mL^−1^, which were rather high for in vitro studies. Indeed, Sales et al.^[^ ^46^ ^]^ found that BMP‐2 concentrations above 5 µg mL^−1^ led to a decrease in hPDSCs adhesion. For C2C12 cells, a plateau was reached from 2.5 to 20 µg mL^−1^. Thus, there may be dose‐dependent responses to BMP‐2 depending on the cell type.

Preclinical studies are a prerequisite to develop bone tissue engineering products, and safety and efficiency should be proved in relevant animal models. Critical‐size bone defects are of particular relevance in view of their close resemblance to clinical situations. Sheep is a particularly good candidate to study bone regeneration in orthopedics because its long bones are of similar size compared to humans' and its bone physiology, biomechanics, stem cells, and response to BMP‐2 are comparable to that in humans.^[^ ^1^, ^47^, ^48^, ^49^, ^50^, ^51^ ^]^ Compared to other long bones such as tibia and femur, metatarsus displays a more hostile environment for bone regeneration as it is encircled by tendons and lacks muscle coverage, a major source of periosteal vascularization, progenitor cells and paracrine stimuli.^[^ ^52^ ^]^ In addition, previous studies from members of our team showed that consistent bone union was achieved in that model when defects were filled with bone autografts, the current standard of care in clinical situations.^[^ ^38^, ^53^ ^]^ Thus, the sheep metatarsal bone appeared as an appropriate model to assess a newly developed bone graft substitute. A similar bone graft substitute made by 3D printing of PLA already proved to be efficient in the more favorable environment which was the minipig mandible.^[^ ^37^ ^]^ Indeed, though the presence of teeth and proximity with saliva induces unfavorable bacteriological conditions, the minipig mandible displays a large amount of well‐vascularized cortico‐cancellous bone with extensive muscle coverage, which creates more conducive conditions for bone regeneration.^[^ ^54^ ^]^ Here, we proved that the bone graft substitute could also regenerate bone in a hostile and load‐bearing environment.

In this study, we compared the efficiency of different scaffold geometries to repair a critical‐size sheep metatarsal bone defect. We designed an implant that could form new bone mimicking the natural architecture of the metatarsal bone, which is made solely of cortical bone. An extensive literature search revealed that there are only three studies investigating pore shape in vivo and all of them were conducted in small animals (2 in rats and 1 in rabbits).^[^ ^55^, ^56^, ^57^ ^]^ Thus, our study provides a new insight by studying bone tissue regeneration in a large animal, that is, sheep.

In our previous study on minipig mandibles,^[^ ^37^ ^]^ we developed a CT‐scan score to qualitatively assess bone regeneration over time. Here, a similar score was used to qualitatively assess bone formation based on X‐rays (Figures 6b and 7b), the initial score being slightly adapted by considering only one type of bone, without distinction between cortical and cancellous bones, which can barely be distinguished on radiographs. We found that this simple score, which can be given by clinicians, agrees well with the quantitative results obtained by analyzing µCT images. Indeed, two main groups can be observed on the graph: on the left, conditions that did not lead to new bone formation and on the right, corresponding to a threshold score of about 1.5, conditions that led to new bone formation (Figure 7e). This finding is of great interest, since it provides an easy and fast qualitative assessment of the amount of newly formed bone. It can be a timesaving predictive tool of the synthetic graft success, without the need to wait for µCT images.

Our study presents some limitations. Indeed, the number of animals used may be considered low. However, it is difficult to propose studies with larger group sizes when working with large animals and it is desired to limit the number of animals used to respect the 3Rs principle. A priori statistical power analysis showed that for an effect size set to 0.5, α set at 0.05, and desired power set at 0.80, 64 sheep would be needed per group to see a statistical difference, which is inconceivable. If keeping the group size at 7, the effect size should be >1.6 in order to reach a power >0.80. A post‐hoc statistical power analysis showed that power was 0.11 when comparing Cubic S + BMP‐2 versus Gyroid S + BMP‐2 (effect size of 0.4); 0.92 for Cubic S + BMP‐2 versus Cubic S w/o BMP‐2 (effect size of 3.2); and 0.28 for Gyroid S + BMP‐2 versus Gyroid S w/o BMP‐2 (effect size of 1.3).

Another limitation is the incomplete loading of implants during the study due to the cast and walking bar maintaining the limb. Since it is known that mechanical constraints are required for efficient bone repair, this incomplete loading may have led to less bone formation. In the present study, we decided not to include mechanical tests in the parameters used to assess the newly formed bone. The rationale for this choice was based on several points. First, mechanical testing is meaningful solely if a clinical healing is observed, which was not the case for all sheep in this study. Furthermore, performing mechanical testing at 4 months would be too early since the osteosynthesis plate is sufficient to maintain mechanical stability. Besides, mechanical testing is destructive and would require to dedicate animals solely for these tests. In this study, we chose to use the explants to perform histological analyses. Furthermore, we noticed that the diameter of the scaffold, which was standardized and similar for all implants, was sometimes smaller than the diameter of the host bone. This led to a misalignment of the scaffold with bone, which might be at the origin of a lower bone formation. Ideally, the dimensions of the scaffold should perfectly match those of the bone defect to favor osseointegration.^[^ ^58^ ^]^ This highlights the necessity to fabricate, in future assays, personalized scaffolds that would be perfectly adapted to each specific defect.

Controlling the BMP‐2 dose is of prime importance in the clinical applications, since it was already shown that a too high BMP‐2 dose can lead to adverse effects.^[^ ^59^ ^]^ The BMP‐2 dose should be carefully adapted to ensure an optimal bone regeneration without side effects. In this study, a BMP‐2 dose of ≈80 µg cm^−3^ of defect was targeted, based on our previous study on minipig mandible,^[^ ^37^ ^]^ where an efficient bone regeneration was found for this dose. BMP‐2 incorporation in 3D scaffolds was found to be higher in the present study compared to the previous one, with BMP‐2 loaded dose of ≈120 µg cm^−3^ of defect. In previous studies in sheep, Yang et al. used a BMP‐2 dose of 400 µg cm^−3^ of defect to repair a 5 cm‐long tibial defect.^[^ ^60^ ^]^ According to them, several studies in preclinical large animal models used BMP‐2 doses from 100 to 800 µg cm^−3^.^[^ ^59^, ^61^, ^62^, ^63^ ^]^ In another study, Maus et al. repaired a trepanation defect in sheep distal femoral epiphysis with a BMP‐2 dose of 200 µg cm^−3^ of defect.^[^ ^64^ ^]^ Similarly, Cipitria et al. repaired a sheep metatarsal critical‐size bone defect using a different architectured material (PCL) combined with a different BMP protein, namely BMP‐7, at a dose of ≈190 µg cm^−3^ of defect.^[^ ^49^ ^]^ Thus, in all these other studies, the BMP‐2 doses were between 1.6‐ and 4‐fold higher than in the present one. The dose of ≈120 µg cm^−3^ represents a notable decrease (12‐fold decrease) in comparison to the 1.5 mg mL^−1^ (1500 µg cm^−3^) loaded into the collagen sponges used in clinics. Such lower BMP‐2 dose delivered via the surface coating of a 3D‐printed scaffold enabled efficient and safe bone regeneration.

We found that a cubic geometry with a mean pore size of ≈870 µm could efficiently regenerate bone in 5/7 cases. Comparatively, the gyroid geometries tested with mean pore sizes of ≈1 mm and ≈805 µm did not regenerate bone so consistently (for Gyroid L, 2/2 scaffolds partially bridged the bone defect and for Gyroid S, 3/7 fully bridged the defect, 1/7 partially bridged it, and 3/7 did not bridge it at all). These results are contrasting with those found by Van Hede et al., who compared cubic (called orthogonal in their study) and gyroid structures in a calvarium rat model with a pore size of 700 µm.^[^ ^55^ ^]^ The different results may be explained by the different implantation sites (metatarsal bone vs calvarium), the animal model used (sheep vs rat), the scaffold material (polymer vs ceramics), and the presence or absence of BMP‐2 (no BMP‐2 was added in Van Hede et al. study). Notably, the implantation in Van Hede et al. was subperiosteal (extremely favorable environment) without any real loss of substance, especially without any critical‐size bone defect.^[^ ^55^ ^]^ Besides pore size and shape, another parameter has been identified by researchers as important for bone regeneration: permeability.^[^ ^65^, ^66^, ^67^ ^]^ The ratio of scaffold surface over volume may also play a role since the higher the ratio will be, the more surface will be available for cells to adhere.^[^ ^68^, ^69^ ^]^ When considering the volume of the defect, surface/volume ratio was the highest for Cubic S (1.8) followed by Cubic‐Gyroid (1.4), Gyroid S (1.35), and Gyroid L (1.1).

Using an in silico modeling approach, Jaber et al.^[^ ^70^ ^]^ recently compared the gyroid and cubic (called strut‐like in their study) scaffold architectures to predict bone formation in the defect, taking into account the influence of mechanical cues and cellular dynamics. Interestingly, they found that the large surface curvatures of the gyroid scaffold resulted in slower tissue formation dynamics and significantly reduced bone regeneration. In addition to the differences in mechanical properties between the two types of scaffolds, they noted differences in the surface‐to‐volume ratio that may explain the superiority of cubic over gyroid. In our study, we also found that Cubic S geometry displayed a higher (1.8‐fold) compressive modulus and (1.6‐fold) compressive strength compared to the Gyroid S scaffold. In addition, the surface to volume ratio for Cubic S (1.8) was higher than the Gyroid S (1.35) geometry. Although these are hypotheses that need to be confirmed in further studies, the differences between the gyroid and cubic geometries in terms of mechanical properties, porosity at the scaffold‐bone interface, and surface‐to‐volume ratio may explain the observed bone regeneration results.

## 4 Conclusion

We designed PLA scaffolds with different internal geometries, fabricated them by FDM, and coated them with a biomimetic polyelectrolyte film delivering BMP‐2 to repair a critical‐size metatarsal bone defect in sheep. Film‐coated PLA loaded with BMP‐2 was proved to be biocompatible both in vitro and in vivo. By tuning scaffold internal geometry, we showed that scaffold geometry influenced BMP‐2 incorporation and bone regeneration. X‐ray scans, µCT scans, and histology proved that scaffolds with cubic pores of ≈870 µm loaded with BMP‐2 at ≈120 µg cm^−3^ led to the formation of new bone without any adverse effects. The new bone formed homogeneously in the longitudinal direction of the bone defect. Notably, the BMP‐2 dose used here was ≈12 fold lower than in the commercially available collagen sponges, and ≈1.6 to 4‐fold lower than comparative studies in large animals. Furthermore, the clinical score given by clinicians on the X‐ray scans acquired during animal follow‐up revealed to be an easy predictive tool of the quantitative assessment of bone volume done by µCT scans. This work opens perspectives for a future personalized treatment of large bone defects in patients, by adapting scaffold shape and size to each patient and precisely controlling the BMP‐2 dose delivered via the film coating of the 3D scaffold.

## 5 Experimental Section

### Materials and Chemicals

Polyethyleneimine (PEI, Sigma, Saint‐Quentin‐Fallavier, France), sodium hyaluronate (HA, Lifecore, Chaska, USA), and poly(l‐lysine) (PLL, Sigma) were dissolved respectively to 5, 1, and 0.5 mg mL^−1^ in a *N*‐(2‐hydroxyethyl)piperazine‐*N*′‐ethanesulfonic acid (HEPES)/NaCl buffer (20 mm HEPES at pH 7.4, 0.15 m NaCl). HEPES and NaCl were purchased from Sigma. 1‐ethyl‐3‐(3‐dimethylaminopropyl)carbodiimide (EDC, Sigma) and *N*‐hydroxysulfosuccinimide (Sulfo‐NHS, Chemrio, Ningbo, China) were dissolved to 30 or 50 mg mL^−1^ for EDC (respectively abbreviated EDC30 and EDC50) and 11 mg mL^−1^ for Sulfo‐NHS in 0.15 m NaCl at pH 5.5.^[^ ^71^ ^]^ BMP‐2 was obtained from InductOs kit (Medtronic, USA). PLL labeled with fluorescein 5‐isothiocyanate (PLL^FITC^) and 5^(6)^‐carboxytetramethylrhodamine *N*‐succinimidyl ester (Rhod) were purchased from Sigma.

Clinical‐grade PLA (cgPLA, Lactoprene 100 m Monofilament, Poly‐Med, Inc, Anderson, USA) was used for BMP‐2 loading experiments and all in vivo experiments and PLA filament of regular‐grade (rgPLA, Verbatim, PLA filament 1.75 mm, Düsseldorfer, Germany) was used for other experiments.

### Design and 3D Printing of PLA Scaffolds

The external shape of the scaffolds was designed using OnShape (http://www.onshape.com). Then, the internal architecture was designed on Ultimaker Cura 4.5 (Ultimaker B.V., Utrecht, Netherlands) by choosing an infill pattern corresponding to the pore shape (either Cubic for cubic opened pores, Zigzag for cubic semi‐closed pores or Gyroid for gyroid pores) and an infill density or infill line distance corresponding to the targeted pore size. This architecture was then modified by directly modifying the code piloting the 3D printer, named G‐code. This G‐code was modified manually and using a homemade Python code. Once designed, the scaffolds were 3D‐printed using an Ender‐3 3D printer (Creality3D, Shenzhen, China). Different types of 2D PLA discs and 3D scaffolds were designed and 3D‐printed depending on the purpose of each specific in vitro or in vivo experiment (Figure 1). For all types of scaffolds, PLA filaments deposited layer‐by‐layer were ≈400 µm in width and ≈200 µm in height. The 3D‐architectured scaffolds had always an external geometry consisting in a cylinder with a differentiated inner ring. Their unit cell was either cubic, gyroid, or a combination of cubic and gyroid. The 2D discs were not architectured.

### Designs of Experiments (DOE)

DOE were performed using a dedicated software (Design‐Expert 12, Stat‐Ease, Inc., Minneapolis, USA) to select the scaffold geometries to implant in a sheep critical‐size metatarsal bone defect (Table S1, Supporting Information). The factors (parameters to be optimized) of the DOE were the infill density and infill pattern of scaffolds. The readouts were the mechanical properties (compressive modulus and compressive strength), the porosity (expressed in %), and the effective pore size. To select the geometries, porosity and mechanical properties were maximized and pore size was targeted to be large enough (>800 µm) to allow adequate vascular and bone ingrowth. This was a factorial design that was randomized, the design type was Plackett Burman, and response modeling was reduced to main effects.

### Film Coating of PLA Scaffolds and BMP‐2 Loading

Prior to film coating, PLA scaffolds were always pre‐wetted in ultrapure water for 24 h. Polyelectrolyte multilayer films were deposited at the surface of 2D PLA discs fabricated for in vitro biocompatibility assays, except the 35.2 mm diameter discs, with a liquid‐handling robot (EVO100, Tecan, Lyon, France) as described by Machillot et al.^[^ ^72^ ^]^ The 35.2 mm diameter PLA discs, PLA discs for in vivo experiments on rats, and 3D PLA mini‐scaffolds and scaffolds were coated using a dip‐coating robot (Dipping Robot DR 3, Riegler & Kirstein GmbH, Potsdam, Germany) as described previously.^[^ ^34^, ^71^ ^]^ Briefly, a first layer of PEI was deposited. Then, 24 alternating layer pairs of PLL and HA were deposited to form a (PLL/HA)_24_ film. Films were subsequently crosslinked using EDC and Sulfo‐NHS as previously described.^[^ ^34^, ^37^ ^]^ EDC50 was used for in vitro experiments except BMP‐2 incorporation assays and EDC30 was used for in vivo experiments and BMP‐2 incorporation tests. Scaffolds were finally rinsed with HEPES/NaCl buffer. BMP‐2 was post‐loaded in the films. For biocompatibility in vitro and biocompatibility and biodegradability in vivo experiments, two different concentrations were used: 30 µg mL^−1^ (low dose, BMP‐2 LD) and 60 µg mL^−1^ (high dose, BMP‐2 HD). For BMP‐2 incorporation in 3D experiments, the BMP‐2 concentration varied between 10 and 50 µg mL^−1^, named hereafter BMP10, BMP30, and BMP50. For bone regeneration experiments, BMP‐2 was post‐loaded in the film at a targeted dose of ≈500 µg per total volume of defect, based on a previous study led on minipigs by the authors.^[^ ^37^ ^]^ The fact that scaffolds had different surfaces and volumes was taken into account and BMP‐2 concentration in the loading solution was adapted consequently. Thus, BMP‐2 concentrations in the loading solutions were 43.2 µg mL^−1^ for Cubic S, 27.9 µg mL^−1^ for Gyroid L, 34.8 µg mL^−1^ for Cubic‐Gyroid, and 38.2 µg mL^−1^ for Gyroid S. BMP‐2 was incubated for 2 h at 37 °C. PLA scaffolds were then rinsed before being dried under a biological safety cabinet. For biocompatibility in vitro experiments, prepared samples were stored in multiwell plates sealed with Parafilm (Sigma‐Aldrich, Saint‐Quentin‐Fallavier, France) and stored at 4 °C. Before beginning the assays, the plates were γ‐sterilized at 25 kGy for 92 h (Gamma Cell 3000 Elan, MDS Nordion Canada). For all other experiments, scaffolds were UV‐sterilized after preparation.

### In Vitro Biocompatibility Assays

Four biocompatibility assays were performed on the 2D PLA discs following ISO 10 993 part 5 guidelines^[^ ^73^ ^]^: i) direct cytotoxicity with contact: 3T3 cells (Balb 3T3, clone A31, ATCC) were cultured in a medium with serum (DMEM and 10% SV, ATCC) and put in direct contact with the biomaterials during 24 h (*n* = 4 for all conditions). A qualitative evaluation was done by microscopy and a quantitative evaluation was done by measuring cell viability with neutral red that targeted lysosomal activity; ii) direct cytotoxicity with extract: L929 cells (NCTL L929, ATCC) were cultured without serum and set in contact for 24 h with extracts and extract dilutions of biomaterials (*n* = 4 for all conditions). Then, a qualitative evaluation was performed by microscopy and a quantitative evaluation was done by measuring cell viability with MTT test (Sigma) that targeted mitochondrial activity. For these two cytotoxicity assays, thermanox (Nunc) was used as negative control and latex as positive control; iii) attachment: 50 000 human mesenchymal stem cells (hMSC, Promocell) were cultured without serum (only 2% SV) and set in contact with the biomaterials in 48‐well microplates (*n* = 3 for all conditions). A quantitative evaluation was done after 15 h by a dosage of lysosomal activity (*p*‐nitrophenyl‐*n*‐acetyl‐betaD‐glucosamide, Sigma‐Aldrich); and iv) proliferation: 5000 hMSC were cultured in mesenchymal stem cell growth medium (Promocell) and set in contact with the biomaterials in 48‐well microplates (*n* = 3 for all conditions). A quantitative evaluation was then performed with Alamar blue test at different time points: 1, 4, 7, 11, and 14 days. For these two latest assays, the control was cells cultured directly on plastic. For each of these assays, four experimental conditions were chosen: bare PLA, film‐coated PLA without BMP‐2, and film‐coated PLA with BMP‐2 loaded at two different BMP‐2 concentrations: BMP‐2 LD and BMP‐2 HD. The dimensions of the discs used for the different assays are specified in Figure 1.

Cytotoxicity (with 3T3 and L929 cells) studies were conducted in order to obtain data in view of a future CE marking file: this assay is mandatory on a regulatory point of view and is requested by the notified bodies. Though they were highly informative, the cytocompatibility studies using hMSC assays were complementary for a technical file, and the authors already obtained data indicating that MSCs can grow on the films (unpublished data).

### In Vivo Subcutaneous Implantation of 2D PLA Discs in Rats for Assessment of In Vivo Biocompatibility and Biodegradability of Polyelectrolyte Films

Following ISO 10993‐2 norm, thirty‐six 8‐week old Wistar rats (Charles River, France) weighing ≈250 g were included in the study. Approval was obtained from the animal ethics committee (*APAFIS#33421‐20211101211184565 v3*). Rats were kept 10 days before the surgery. Before anesthesia, perioperative analgesia was implemented using buprenorphine at 0.1 mg kg^−1^ by intraperitoneal injection. Animals were then anesthetized via an induction cage (5% isoflurane and 2 L min^−1^ oxygen), and the anesthesia was maintained using a mask (2.5% isoflurane and 2 L min^−1^ oxygen). An ophthalmic gel (Ocrygel, TVM) was used during anesthesia and put in place before animal clipping. The animals were placed in prone position before clipping and disinfecting their back skin using chlorinetetracycline. Hypothermia was avoided by heating the induction cages and the surgical plan at 37 °C using a heating mat. Following ISO 10993–6 recommendations, the PLA discs of 12 mm in diameter and 0.4 mm in height (Figure 1) were implanted subcutaneously. For that, four incisions of 1 cm were made in the back region and near the flanks using an 11 mm blade. A subcutaneous detachment was made with Metzembaum scissors. A prepared PLA disc was placed in the created pocket. The created pockets were not interconnected. The wound was then closed using intradermal suture with a 4‐0 monofilament (polyglecaprone 25). After surgery, analgesia was maintained when necessary by subcutaneous injection of buprenorphine (0.02 mg kg^−1^). During recovery from anesthesia and until their awakening, the rats were placed in heated cages. A qualified technician clinically evaluated the animals every day, except during the weekend, for the first 7 days after surgery. After the first 7 days, animals were clinically evaluated once a week. Their general condition, feeding, watering, cleaning, morbidity, and mortality was observed and taken care of. Animals were weighed once a week until euthanasia. The study was composed of 4 experimental groups: bare PLA, film‐coated PLA without BMP‐2, and film‐coated PLA with BMP‐2 loaded at two different BMP‐2 concentrations: BMP‐2 LD and BMP‐2 HD. All experimental groups were studied at three time points: day 7, 28, and 48 (D7, D28, D48), with *n* = 3 rats for each experimental condition at each time point. The animals were euthanized by pentobarbital intraperitoneal injection followed by an injection of Exagon (1 mL kg^−1^) combined with lidocaine (10 mg mL^−1^). The materials were removed with surrounding tissues and samples from spleen, kidney, brain, heart, and liver. All these specimens were preserved and stored in 4% paraformaldehyde at 4 °C until analysis.

These in vivo experiments were performed for similar reasons than the in vitro biocompatibility experiments: The authors aimed at obtaining standardized data regarding inflammatory response with the final biomaterial in order to meet the regulatory requirements. The authors already obtained data showing biocompatibility in a bone defect in minipigs.^[^ ^37^ ^]^ However, these previous experiments were not performed following the methods recommended by the ISO 10 993 standard.

### Histology and Histomorphometry of 2D PLA Discs

Specimens were fixed in 4% paraformaldehyde formalin (Antigenfix, Microm Microtech, France) with stirring for 24 h. Dehydration of specimens was conducted in ascending alcohol baths (Absolute ethanol ≥ 99.8%, VWR Chemicals), then in toluene (Toluene N/A ≥ 99%, VWR Chemicals), followed by paraffin (Paraplast X‐TRA, LEICA Biosystems) impregnation using a LEICA TP1020. They were then embedded in paraffin using a LEICA EG1150H embedding platform. Sections ≤ 6 µm were cut using a microtome (LEICA RM2255) and stained with Hematoxylin Erythrosine Saffron staining (HES). They were then analyzed with a microscope (Nikon NiU). Histomorphometry was conducted using a dedicated software (NIS Elements D). To quantify the film still present at the implant surface after explantation and sample processing, the percentage of the film remaining at the PLA discs surface was calculated based on the measurements of the disc perimeters and of the film length along the disc surface.

### Study of BMP‐2 Loading in 3D

To assess the effect of pore size and shape on BMP‐2 loading inside different 3D architectures, cylinders of 8 mm in height and 14 mm in diameter, hereafter called 3D mini‐scaffolds, were designed and 3D‐printed (Figure 1) with cgPLA. Five scaffold architectures were used: Cubic S, Cubic L, Gyroid S, Gyroid L, and Cubic‐Gyroid. After coating with the polyelectrolyte film, BMP‐2 was loaded at BMP10, BMP30, and BMP50 (*n* = 3 3D mini‐scaffolds per geometry and per BMP‐2 concentration). The homogeneity of BMP‐2 loading inside the film‐coated 3D mini‐scaffolds was assessed using a BMP‐2 loading solution containing 5% of BMP‐2^Rhod^ and a fluorescence macroscope (Macrofluo Z16 Apo, Leica Microsystems, Wetzlar, Germany) with a 0.8× objective. BMP‐2 loading was quantified using a fluorescence spectrometer (Tecan Spark, Tecan Lyon, France) for BMP10 (excitation at 535 nm, bandwidth 25 nm; emission at 595 nm, bandwidth of 35 nm; number of flashes was set at 30 and the integration time at 40 µs) and a UV–vis spectrophotometer (Cary 60, Agilent Technologies, Inc., Santa Clara, USA) for BMP30 and BMP50. The amount of loaded BMP‐2 was measured by quantifying the initial concentration of BMP‐2 in the loading solution and the remaining BMP‐2 concentration after loading in the 3D mini‐scaffolds. The amount of BMP‐2 loaded was calculated as the difference between these two concentrations multiplied by the volume of the BMP‐2 loading solution.

### Characterization of Scaffolds and Film Coating

Two types of PLA filaments were used in this study: rgPLA, which was used for all in vitro experiments except the BMP‐2 incorporation tests in 3D and cgPLA, which was used for all in vivo experiments and the BMP‐2 incorporation tests in 3D. The differences between the two filaments, notably their crystallinity that influences the degradation rate, were investigated by ATR‐FTIR and SAXS. A Ge crystal, Perkin‐Elmer spectroscope, and Spectrum software were used for ATR‐FTIR. Pieces of PLA filaments were used for the measurements, and background was subtracted for every measurement. Spectra were collected in the range of 4000 to 600 cm^−1^, at a 2 cm^−1^ resolution and with 16 scans. For the SAXS acquisition, PLA filaments were cut and the pieces of filaments were arranged in order to create 1 cm^2^ squares. Acquisitions were made in *θ*/2*θ* reflection mode and cobalt radiation (*λ* = 0.17 903 nm) was used.

3D mini‐scaffolds and 3D scaffolds were imaged by µCT using a VivaCT 40 (SCANCO Medical AG, Brüttisellen, Switzerland) to quantify their porosity and surface area. The acquisition parameters were set at 70 kV with an intensity of 114 µA, an isotropic voxel size of 76 µm, and integration time of 100 ms. For 3D scaffolds, four geometries were characterized: Cubic S, Gyroid S, Gyroid L, and Cubic‐Gyroid. 3D mini‐scaffolds were imaged using an Ultra 55 SEM (Zeiss, Oberkochen, Germany). Prior to imaging, 3D mini‐scaffolds were metallized with platinum. Pore sizes were evaluated at 10 kV with a secondary electron (SE2) detector. The mechanical properties of the 3D scaffolds were measured by performing uniaxial compressive tests with a traction machine (MTS Systems Corporation, Eden Prairie, USA). A10 kN load cell at a speed of 1 mm s^−1^ was used. Tests were performed until 10% of deformation was reached. Tests were done in triplicate for each scaffold geometry. The compressive strength and compressive modulus (both expressed in MPa) were deduced from the stress–strain curves. The compressive strength was defined as the maximum stress withstood by the scaffolds and the compressive modulus was defined as the slope of the linear (elastic) part of the stress–strain curve.

To image the film deposited onto the 3D scaffolds, the last deposited layer was labeled with FITC (PLL^FITC^).^[^ ^33^, ^37^ ^]^ The scaffolds were then stored in 0.15 m NaCl until imaging using a fluorescence macroscope (Macrofluo Z16 Apo, Leica Microsystems, Wetzlar, Germany) with a 0.8× objective.

### In Vivo Sheep Metatarsal Critical‐Size Bone Defect

Twenty‐four mature female Pré‐Alpes sheep, mean age of 36.2 months (21.2–61.7 months) and weighing 63 kg (46.5–79 kg) were included in this study, which was approved by the animal ethics committee (*APAFIS#20287‐2019041715086916 v2*). The animals were obtained from “Les élevages Christian Lebeau” (Gambais, France). Animal housing and care were carried out using procedures previously described.^[^ ^47^ ^]^ The pre‐surgical (notably anesthesia) and surgical procedures were performed as previously described.^[^ ^38^ ^]^ Briefly, a 25‐mm long mid‐diaphyseal osteotomy was performed in the left metatarsal bone with full periosteal removal. This defect was stabilized with an osteosynthesis plate (3.5 Dynamic Compression Plate, Synthes) and cortical screws of 3.5 mm in diameter. An implant was then inserted into the defect. Cerclages using two 2/0 polydioxanone sutures (PDS I, Ethicon) were used around the implant and plate to enhance stability at the replacement site. The wound was then closed. A cylindrical cast including a 5‐mm‐diameter steel walking bar was placed around the operated hind limb of each sheep. Aftercare was conducted as previously described.^[^ ^38^ ^]^ The animals were followed up for 4 months. A veterinarian clinically evaluated the animals every day.

Each implant was randomized, implanted, and analyzed in a blind manner using X‐ray scans. In the preliminary experiment, three scaffold geometries were tested (*n* = 2): Cubic S, Gyroid L, and Cubic‐Gyroid, all coated with the biomimetic film and loaded with BMP‐2. In the main experiment, two selected conditions were further studied in larger groups to perform statistical analysis. The Cubic S geometry loaded with BMP‐2 (*n* = 5), hereafter referred to as Cubic S + BMP‐2, was kept and the Gyroid S geometry loaded with BMP‐2 (*n* = 7), referred to as Gyroid S + BMP‐2, was introduced in order to compare two scaffold geometries with a similar pore size (870 and 805 µm for Cubic S and Gyroid S, respectively). Two negative controls were added for each geometry: film‐coated scaffolds without BMP‐2, referred to as Cubic S w/o BMP‐2 and Gyroid S w/o BMP‐2 (see **Table** **1** for all experimental conditions). Additional groups using bone autografts (standard care) or defects left empty were not added in the present study, as the authors previously provided evidence of consistent occurrence of bone union using autologous bone grafting and absence of union in an empty defect in the same model in sheep.^[^ ^53^ ^]^ However, data from bone autografts were added using a previous study led by the authors. It was a way to reduce the number of animals used, which was in line with the 3Rs principle on animal research. The two Cubic S + BMP‐2 scaffolds implanted in the preliminary experiment were included in the analysis of the main experiment to reach *n* = 7 without the need to use more sheep. Sheep were euthanized after 4 months by overdose of barbiturate. The cast of each sheep was removed and the left metatarsus was excised, radiographed, and fixed in 4% paraformaldehyde under mild shaking for 2 weeks.

**Table 1:** Experimental conditions and total number of sheep studied per experimental group. For the preliminary experiment, *n* = 2 for each condition. For the main experiment, *n* = 2 Cubic S w/o BMP‐2 and Gyroid S w/o BMP‐2 were added as negative controls (no BMP‐2), *n* = 5 Cubic S + BMP‐2 were added, and *n* = 7 Gyroid S + BMP‐2 were added. Film was crosslinked at EDC30 and loaded with BMP‐2. In this table, the first number on the left of the “+” refers to the conditions of the preliminary experiment, while the second number refers to the main experiment.

Scaffold geometry	Condition	
	Film no BMP‐2	Film + BMP‐2
Cubic S	0 + 2	2 + 5
Gyroid S	0 + 2	0 + 7
Gyroid L	–	2 + 0
Cubic‐Gyroid	–	2 + 0

### Analysis of Bone Growth within 3D Scaffolds

Qualitative assessment of bone formation was done by acquiring X‐ray scans each month, and after bone resection at 4 months post‐surgery. These radiographs were acquired using an EvolutX FP veterinary radiograph system (Medec Loncin, Belgium) under anesthesia. The acquisition parameters were set to 69 kV and 12.8 mA s. A score, adapted from the one previously developed by the authors, was used to qualify bone formation:^[^ ^37^ ^]^

(1) $$S=\frac{2F+2H-E}{4}$$

This score is composed of three criteria: i) the percentage of filling of the porous implant (*F*), ii) the homogeneity of the newly formed bone (*H*), and iii) the amount of bone outside of the implant, that is, “ectopic” bone (*E*). Each criterion was evaluated by five clinicians in a blind manner using a score between 0 and 4. The score was represented as the mean score ± standard deviation (SD) of the scores given by each evaluator independently.

After fixation of the explants, the osteosynthesis plates were removed. A piece of tissue around the bone site defect (extra length of 1 cm on each side) was collected and stored in water. Specimens were then imaged with a high‐resolution µCT (Skyscan1172, Bruker) with the following settings: 90 kV source voltage, 279 mA source current, 17.7 µm pixel size, 0.3° rotation step, 420 ms exposure time, a frame averaging of 8, and aluminum‐copper filters. The images were scanned and the bone defect was reconstructed by means of NRecon software (V1.7.4.6, Bruker). A 3D reconstruction of samples was made using CT Vox software (CT Vox v.3.3.1, Bruker). The reconstructed images were then imported into Dragonfly software (ORS Inc., Canada) for quantitative analyses with binarization threshold for bone determined by Otsu's method. For quantification of the total newly formed bone between edges, data were treated with a volume of interest corresponding to a cylinder centered in the middle of the defect with a 25 mm length. The VOI was also divided into three equal parts corresponding to the proximal, central, and distal areas. These results were compared with those obtained using fragmented iliac crest bone autografts, following the same surgical protocol and using the same image acquisition and analysis parameters as in Decambron et al.^[^ ^74^ ^]^

### Undecalcified Histology

Specimens were dehydrated and embedded in methyl methacrylate resin as described in Viateau et al.^[^ ^38^ ^]^ The embedded specimens were cut along the metatarsal axis using a circular saw (200–300 µm, Leitz 1600, Leica Biosystems) and a central section (closest to the mid‐sagittal plane) and a peripheral section were selected for histological analysis, ground down to 100 µm thick, polished, and stained with Stevenel blue and Van Gieson picrofushin.

### Statistical Analysis

Data were always used as obtained by the different apparatus. There was no pre‐processing of the data. Data were expressed as mean ± SD. Sample size was given for each graph of the figures. It depended on the type of assay. Origin 2020 (OriginLab Corporation) and Excel (Microsoft Office) were used for all graphical and statistical analyses. Non‐parametric data were presented by median and interquartile range. Differences between groups were assessed by analysis of variance (ANOVA) and Bonferroni post‐hoc analysis or Student's *t*‐test for parametric data, and by Kruskal–Wallis ANOVA with Dunn's test, Dunnett's test, and Mann–Whitney *U* test for non‐parametric data. Two‐way ANOVA was used to assess differences when two independent variables were used. G* power 3.1 (Heinrich‐Heine‐University) was used for statistical power analysis. Differences between groups at *p* < 0.05 (*) and *p* < 0.01 (**) were considered as significant.

## Conflict of Interest

The authors declare no conflict of interest.

## Author Contributions

S.S., C.M., P.M., C.D., S.R., and J.V. are co‐second authors. J.V., M.R., H.G., and H.E.‐H. are co‐third authors. C.G.:Conceptualization, methodology, software, validation, formal analysis, investigation, resources, data curation, writing—original draft, writing—review and editing, visualization. S.S.: Formal analysis, investigation, data curation, visualization. CM: Conceptualization, methodology, validation, formal analysis, investigation. P.M.: Conceptualization, methodology, software, validation, investigation, resources. C.D.: Conceptualization, methodology, validation, formal analysis, investigation. S.R.: Conceptualization, methodology, validation, formal analysis, investigation. J.Vi.: Investigation. J.Vo: Methodology, validation, investigation, resources. M.R.: Conceptualization, methodology, validation, formal analysis, investigation. H.G.: Investigation, resources. H.E.‐H.: Methodology, investigation, data curation. A.D.: Investigation. V.J.: Validation, resources, writing—review and editing, supervision, project administration, funding acquisition. L.B.: Supervision. G.B.: Conceptualization, methodology, validation, formal analysis, resources, writing—review and editing, supervision, funding acquisition. M.D.: Writing—original draft, writing—review and editing, supervision, project administration, funding acquisition. M.M.: Conceptualization, methodology, validation, formal analysis, investigation, resources, data curation, supervision, project administration, funding acquisition. V.V.: Conceptualization, methodology, validation, formal analysis, investigation, resources, data curation, writing—review and editing, supervision, funding acquisition. D.L.‐A.: Conceptualization, methodology, validation, formal analysis, resources, data curation, writing—original draft, writing—review and editing, visualization, supervision, project administration, funding acquisition. C.P.: Conceptualization, methodology, validation, writing—original draft, writing—review and editing, supervision, project administration, resources, funding acquisition.

## Supporting information

Supporting Information

## Data Availability

The data that support the findings of this study are available from the corresponding author upon reasonable request.
